# Decreased deiodinase activity after glucose load could lead to atherosclerosis in euthyroid women with polycystic ovary syndrome

**DOI:** 10.1007/s12020-019-01913-0

**Published:** 2019-04-03

**Authors:** Agnieszka Adamska, Anna Krentowska, Agnieszka Łebkowska, Justyna Hryniewicka, Monika Leśniewska, Marcin Adamski, Irina Kowalska

**Affiliations:** 1 Department of Endocrinology, Diabetology and Internal Medicine, Medical University of Białystok, Białystok, Poland; 2 Department of Internal Medicine and Metabolic Diseases, Medical University of Białystok, Białystok, Poland; 3 Department of Reproduction and Gynecological Endocrinology, Medical University of Białystok, Białystok, Poland; 4 Faculty of Computer Science, Bialystok University of Technology, Białystok, Poland

**Keywords:** Thyroid, PCOS, Lipids

## Abstract

**Objective:**

Glucose and lipid disturbances, as well as higher tendency to atherosclerosis, are observed in women with polycystic ovary syndrome (PCOS). Thyroid hormones action has long been recognized as an important determinant of glucose and lipid homeostasis. Some studies suggest that even in euthyroid subjects, thyroid function may affect atherosclerosis risk factors. The aim of this study was to evaluate the relationships between thyroid hormonal status and glucose and lipid profile before and after oral glucose tolerance test (OGTT) in PCOS women in comparison to the control group.

**Patients and methods:**

The study group included 98 women—60 women with PCOS and 38 women matched for age and BMI as a control group. OGTT with estimation of plasma glucose and lipids, as well as serum insulin and thyroid hormones (TH) concentrations was performed. Activity of peripheral deiodinases at baseline (SPINA-GD1) and at the 120 min of OGTT (SPINA-GD2) was calculated according to the formula by Dietrich et al. as a measure of T4–T3 conversion efficiency. Delta GD was estimated as SPINA-GD1–SPINA-GD2, and delta fT3 was calculated as a difference between fT3 before and after OGTT.

**Results:**

We did not find differences in TH, SPINA-GDs, and plasma lipid concentrations between PCOS and control group before and after OGTT. Glucose load resulted in a decrease of level TSH, TC, TG, HDL-C, and LDL-C concentrations in women with PCOS, as well as in the control group (all *p* < 0.05). We found that GD (*p* = 0.01) and serum fT3 concentration (*p* = 0.0008) decreased during glucose load only in the PCOS group. We observed a positive relationship between delta fT3 and plasma TG concentration (*r* = 0.36, *p* = 0.004), delta GD and plasma TG concentration after glucose load (*r* = 0.34, *p* = 0.007), only in the PCOS group. We also found negative relationship between SPINA-GD2 and plasma TC concentration (*r* = −0.29, *p* = 0.02) after glucose load and positive relationship between delta GD and insulin at the 60 min of OGTT (*r* = 0.29, *p* = 0.02), only in the PCOS women.

**Conclusions:**

These data showed insufficient conversion of fT4 to fT3, as well as a relationship of SPINA-GDs with insulin, TC and TG in PCOS women after glucose load. It may suggest that disturbances in deiodinase activity after glucose load might promote atherosclerosis in PCOS women.

## Introduction

Polycystic ovary syndrome (PCOS) is recognized as the most common endocrinological disorder and affects up to 20% of premenopausal women [1]. PCOS is characterized by clinical and/or biochemical hyperandrogenism, ovulatory dysfunction, and characteristic image of the ovaries in ultrasound [2]. Moreover, PCOS is characterized not only by symptoms associated with reproductive system but also by metabolic dysfunction, which could be linked with an increased risk for cardiovascular disease (CVD) [3]. The higher predisposition to CVD is associated with higher tendency to abdominal obesity, early onset of type 2 diabetes mellitus, hypertension, as well as more prevalent dyslipidemia in PCOS women [4, 5]. The most common pattern of dyslipidemia in PCOS women is a so-called atherogenic lipoprotein phenotype, which is characterized by hypertriglyceridemia, low serum levels of high-density lipoprotein cholesterol (HDL-C) and also observed increased values of low-density lipoprotein cholesterol (LDL-C) [4].

It is well established that thyroid hormones (TH) are responsible for activation of genes encoding proteins with crucial role in maintaining lipid metabolism. There is an inverse relationship between thyroid status and serum lipid concentrations [6]. TH regulate the activity of several key enzymes involved in the lipoprotein transport, e.g. cholesteryl-ester transfer protein (CETP) and hepatic lipase (HL), and thus modulate the distribution of HDL-C [6]. TH may exert impact on the synthesis and degradation of LDL-C. The promoter of the LDL receptor gene contains a triiodothyronine (T3) responsive element which modulates gene expression of the LDL-C receptor resulting in an increase of LDL-C clearance [7].

The upkeep of normal levels of circulating TH is a crucial function of the hypothalamus–pituitary–thyroid axis (HPT). Thyroid hormone homeostasis is controlled by a highly specific system in which deiodinases (DIOs) represent a critical step in tissue-specific modulation of the hormonal message. There are three types of iodothyronine deiodinases: type 1 (DIO1), type 2 (DIO2), and type 3 (DIO3). DIO1 is mainly expressed in the liver, kidneys, thyroid gland, white adipose tissue and pituitary, whereas DIO2 is mostly present in the brown adipose tissue, placenta, pituitary, and muscle, and DIO3 is expressed in the placenta, central nervous system, skin, and fetal liver [8, 9]. Deiodination is a process by which minimally active thyroxine (T4) is transformed into a ligand for thyroid hormone receptors triiodothyronine (T3) [10]. Production of T3 is catalyzed by DIO1 and DIO2 through the outer ring deiodination of the prohormone T4. In contrast, DIO3 catalyzes the inner ring deiodination, leading to inactivation of T4 into reverse triiodothyronine (rT3) [11]. Various methods for the determination of deiodinase activity in serum have been described. The level of their expression can be determined using polymerase chain reaction method, as well as by means of available enzyme-linked immunosorbent assays (ELISA) [12]. Recently, Dietrich et al. presented a mathematical model for assessing the sum of peripheral deiodinase activity (DIO1 and DIO2), which have been validated in numerous clinical trials [13–15]. The aforementioned model uses the measurement of serum thyroid hormone concentrations (TSH, fT3, and fT4) and constant parameters of binding, distribution, and elimination of plasma proteins [15].

Currently, there are no studies assessing the relationship of serum deiodinase activity with lipid profile and glucose concentrations in PCOS group. Therefore, this study was undertaken to evaluate the relationships between thyroid hormonal status and serum lipid concentrations before and after oral glucose tolerance test (OGTT) in PCOS women compared to the control group.

## Subjects and methods

### Subjects

The study group included 98 women—60 women with PCOS and 38 women matched for age and BMI as a control group. PCOS was diagnosed according to the 2003 Rotterdam ESHRE/ASRM PCOS Consensus Workshop Group diagnostic criteria, which have been described previously [16]. Women with PCOS were recruited from the Department of Endocrinology, Diabetology and Internal Medicine, Medical University of Białystok and among students. Students were enrolled as control subjects. All women were non-smoking. Clinical examination, anthropometric measurements, OGTT with 75 g of glucose, were performed as previously described [16]. Thyroid ultrasound was performed to evaluate the structure and volume of the thyroid gland. Exclusion criteria were: thyroid disorders, e.g. any changes in the function of the thyroid gland (hypothyroidism, hyperthyroidism), morbid obesity, CVD, hyperlipidemia; other causes of irregular menstrual cycles and/or androgen excess (i.e., hyperprolactinemia, Cushing’s syndrome, late-onset congenital adrenal hyperplasia, or diseases of the adrenal glands, pregnancy, and breastfeeding); type 1 or type 2 diabetes; chronic or acute infection (within the previous 30 days); any other serious medical problem, hormonal contraception and/or anti-androgen therapy (within the previous 6 months). Moreover, participants taking any medications (e.g. drugs affecting lipid and glucose metabolism, radioactive iodine, levothyroxine) were also excluded from the study. All analyses were carried out after an overnight fast. The studies were performed in the PCOS group 3–5 days after a spontaneous menses or independent of cycle phase in the presence of amenorrhea. In the control group, the studies were performed during the early follicular phase (3–5 days) of their menstrual cycles. Written consent has been obtained from each patient or subject after full explanation of the purpose and nature of all procedures used. The study protocol was approved by the Ethics Committee of the Medical University of Bialystok and was concordant with the Declaration of Helsinki.

### Biochemical analyses

Plasma glucose level was measured immediately by the enzymatic reference method with hexokinase (Cobas c111, Roche Diagnostic Ltd., Switzerland). Serum insulin concentration was assayed by immunoradiometric method (DIAsource ImmunoAssays S.A., Belgium). The minimum detectable concentration was 1 µIU/mL and the intra-assay and inter-assay coefficients of variation (CVs) were below 2.2% and 6.5%, respectively. In this method, human and animal proinsulins present no cross-reactions. Plasma total cholesterol (TC), HDL-C, and triglicerydes (TG) were assessed by enzymatic methods using commercial kits produced by ANALCO-GBG, Poland. Plasma LDL-C was calculated according to the Friedewald’s formula. Serum FSH, LH, prolactin, and total testosterone concentrations were measured by immunoradiometric method (DIAsource ImmunoAssays S.A., Belgium). Serum sex hormone-binding globulin (SHBG) was measured by immunoradiometric assay (ZenTech, Angleur, Belgium). Free androgen index (FAI) was calculated as serum total testosterone (nmol/L) × 100/SHBG (nmol/L) ratio [17]. Serum TSH concentration was estimated by immunoradiometric method. Serum fT3 and fT4 concentrations were detected by radioimmunoassay kits (DIAsource ImmunoAssays S.A., Belgium). The sensitivity and CVs for all of these assays were identical, as reported previously [18]. We calculated delta fT3 as a difference between fT3 before and after OGTT.

The homeostasis model assessment of insulin resistance (HOMA-IR) was calculated according to the formula: (fasting insulin (µIU/mL) × fasting plasma glucose (mmol/L))/22.5 [19]. We calculated the sum (SPINA-GD) of peripheral deiodinases activity (DIO1 and DIO2) from equilibrium levels of TSH, fT4, fT3 and estimated constant parameters for plasma protein binding, dissociation, and hormone kinetics according to the equation of Dietrich et al. [20] at baseline (SPINA-GD1) and at the 120 min of OGTT (SPINA-GD2). Delta GD was calculated as SPINA-GD1−SPINA-GD2.

### Ultrasonography of the thyroid gland

Ultrasound of the thyroid gland was made with a 7.5 MHz linear transducer (Philips HD5 Diagnostic Ultrasound System, Bothell, Washington, USA, Neusoft Park, Hun Nan Industrial Area, Shenyang 110179, China). Thyroid volume was calculated using the equation: (length × width × thickness of the lobes) × 0.479 [21].

### Statistical analysis

Statistical analyses were performed using the STATISTICA 10.0 software. Differences between the groups were evaluated with non-parametric Mann–Whitney *U*-test. Wilcoxon signed-rank tests were used to compare estimated variables at baseline and after glucose load in PCOS women and control group. The relationships between variables were evaluated using Spearman’s rank correlation. The level of significance was accepted at *p* < 0.05.

## Results

The clinical and biochemical characteristics of the studied groups are shown in Table 1. The PCOS and control group did not differ in age, anthropometric indices and plasma glucose, lipids, and serum TSH, fT3, and fT4 concentrations at the 0 and 120 min of OGTT, as well as volume of thyroid gland (all *p* > 0.05). We found higher glucose and insulin concentration at the 60 min of OGTT in the PCOS group in comparison to the control group (*p* = 0.004, *p* = 0.09, respectively). Fasting insulin and HOMA-IR were higher in the PCOS women as compared to the control group (*p* = 0.00002, *p* = 0.00002, respectively). We did not observe differences in the estimated SPINA-GD1, SPINA-GD2, as well as delta GD between studied groups (all *p* > 0.05) (Table 1).

**Table 1:** Clinical and biochemical characteristics of the studied groups

	Control group (*n* = 38)	PCOS (*n* = 60)
Age (years)	26.7 ± 3.8	25.6 ± 4.3
BMI (kg/m^2^)	22.9 ± 3.2	24.5 ± 4.3
Waist circumference (cm)	79.9 ± 9.8	84.1 ± 13.0
Hip circumference (cm)	99.9 ± 7.9	99.2 ± 10.0
Follicle-stimulating hormone (IU/l)	5.7 ± 2.0	4.9 ± 1.5
Luteinizing hormone (IU/l)	3.9 ± 1.4	4.8 ± 2.9
LH/FSH ratio	0.72 ± 0.3	1.09 ± 0.8*
Total testosterone (ng/ml)	0.62 ± 0.1	0.75 ± 0.2*
SHBG (nmol/l)	67.8 ± 39.8	53.7 ± 31.6*
FAI	4.2 ± 2.7	6.8 ± 4.9*
Prolactin (ng/ml)	12.6 ± 8.8	12.2 ± 6.6
Glucose 0′ OGTT (mg/dl)	93.5 ± 8.0	93.8 ± 8.1
Glucose 60′ OGTT (mg/dl)	102.8 ± 24.6	122.5 ± 38.5*
Glucose 120′ OGTT (mg/dl)	94.8 ± 18.9	97.8 ± 23.0
Insulin 0′ OGTT (μIU/ml)	8.6 ± 2.4	10.8 ± 4.8*
Insulin 60′ OGTT (μIU/ml)	51.3 ± 36.1	72.5 ± 46.7*
Insulin 120′ OGTT (μIU/ml)	33.9 ± 27.8	41.0 ± 30.5
HOMA-IR	2.0 ± 0.6	2.6 ± 1.2*
Total cholesterol (mg/dl)	176.2 ± 24.8	177.4 ± 29.6
HDL-cholesterol (mg/dl)	68.3 ± 16.2	68.6 ± 15.9
LDL-cholesterol (mg/dl)	94.8 ± 21.5	93.8 ± 25.0
TG (mg/dl)	64.8 ± 26.6	74.8 ± 33.6
Total cholesterol at 120 min OGTT (mg/dl)	163.3 ± 29.2^#^	165.2 ± 26.4^#^
HDL-cholesterol at 120 min OGTT (mg/dl)	61.6 ± 15.6^#^	62.6 ± 17.4^#^
LDL-cholesterol at 120 min OGTT (mg/dl)	90.0 ± 22.4^#^	90.1 ± 22.0^#^
TG at 120 min OGTT (mg/dl)	58.0 ± 26.5^#^	61.8 ± 27.8^#^
TSH (uIU/ml)	2.373 ± 0.641	2.182 ± 0.773
fT4 (ng/dl)	1.363 ± 0.2	1.339 ± 0.196
fT3 (pg/ml)	2.785 ± 0.508	2.852 ± 0.612
TSH at 120 min OGTT (μIU/ml)	1.645 ± 0.415^#^	1.569 ± 0.536^#^
fT4 at 120 min OGTT (ng/dl)	1.345 ± 0.206	1.341 ± 0.205
fT3 at 120 min OGTT (pg/ml)	2.678 ± 0.484	2.691 ± 0.481^#^
SPINA-GD1 (nmol/s)	22.9 ± 4.6	23.6 ± 5.3
SPINA-GD2 (nmol/s)	22.2 ± 4.6	22.2 ± 5.1^#^
SPINA-GD1−SPINA-GD2	0.6 ± 5.0	1.4 ± 4.3
VT	11.5 ± 4.6	11.0 ± 3.2

Data are presented as mean ± SD. Differences between the groups are derived from non-parametric Mann–Whitney *U* test

*BMI* body mass index, *TG* triglycerides, *OGTT* oral glucose tolerance test, *FSH* follicle-stimulating hormone, *LH* luteinizing hormone, *FAI* free androgen index, *SHBG* sex hormone-binding globulin, *HOMA-IR* homeostasis model assessment of insulin resistance, *TSH* thyroid‑stimulating hormone, *fT4* free thyroxine, *fT3* free triiodothyronine, *SPINA-GD1* sum activity of activating peripheral deiodinases at baseline, *SPINA-GD2* sum activity of activating peripheral deiodinases at the 120 min OGTT, *VT* volume of thyroid gland

*p < 0.05 in PCOS women vs. control group, ^#^*p* < 0.05 vs. the baseline state

Women with PCOS had higher LH/FSH ratio (*p* = 0.01), serum concentrations of total testosterone (*p* = 0.02), FAI (*p* = 0.003), and lower serum concentration of SHBG (*p* = 0.02), compared to the control group (Table 1).

Glucose load resulted in a decrease of TSH, TC, TG, HDL-C, LDL-C levels in women with PCOS, as well as in control group (all *p* < 0.05) (Table 1).

**Fig. 1:**
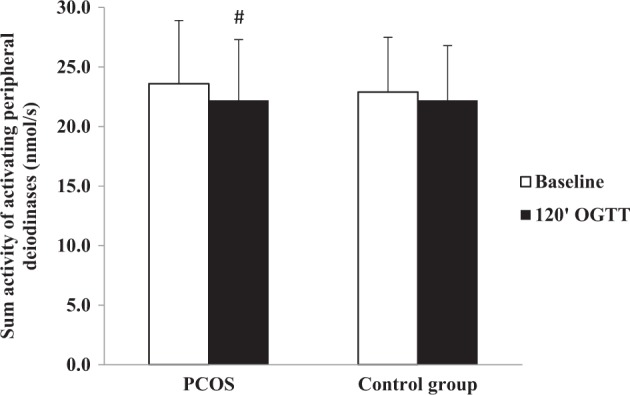
Sum activity of activating peripheral deiodinases at baseline and at the 120 min of oral glucose tolerance test in the PCOS and control group women. #*p* < 0.05 vs. the baseline state

We found that SPINA-GD (*p* = 0.01) and serum fT3 concentration (*p* = 0.0008) decreased after glucose load only in the PCOS group (Fig. 1).

We observed a relationship between SPINA-GD1 and BMI (*r* = 0.29, *p* = 0.02) only in PCOS women. We also found an association between fT3/fT4 ratio and BMI (*r* = 0.28, *p* = 0.02) in this group.

We observed a positive relationship between serum insulin concentration at the 60 min of OGTT and delta GD (*r* = 0.29, *p* = 0.02) in PCOS women. We did not observe correlations of SPINA-GD1, SPINA-GD2 and delta-GD with plasma glucose concentrations at baseline and at the end of OGTT, nor with HOMA-IR (all *p* > 0.05).

We also found a positive relationship between HOMA-IR and plasma TG concentration (*r* = 0.36, *p* = 0.02), and a negative relationship between HOMA-IR and plasma HDL-C concentration (*r* = −0.33, *p* = 0.03), only in the PCOS group.

We found a negative association between SPINA-GD1 and baseline plasma TC concentration (*r* = −0.34, *p* = 0.006), SPINA-GD2 and baseline plasma TC (*r* = −0.40, *p* = 0.001), SPINA-GD2 and plasma TC concentration at the 120 min of OGTT (*r* = −0.29, *p* = 0.02), only in PCOS group. We also observed a negative relationship between SPINA-GD2 and baseline LDL-C concentration (*r* = −0.30, *p* = 0.01) and a positive relationship between delta GD and plasma TG concentration after glucose load (*r* = 0.34, *p* = 0.007), only in PCOS women (Table 1).

We observed a negative correlation between baseline plasma TC concentration and fT3/fT4 ratio (*r* = −0.36, *p* = 0.003), baseline plasma LDL-C concentration and fT3/fT4 ratio (*r* = −0.27, *p* = 0.03), and plasma TC concentration and fT3/fT4 ratio (*r* = −0.3, *p* = 0.01) at the 120 min of OGGT, as well as between delta fT3 and plasma TG concentration at the 120 min of OGTT (*r* = 0.36, *p* = 0.004), only in PCOS.

## Discussion

The main finding of the present study is an observation that activity of peripheral DIOs and, consequently, serum fT3 concentration decreased during glucose load in euthyroid PCOS women compared to age-matched, BMI-matched, and thyroid volume-matched non-PCOS women.

It should be emphasized that although we observed a significant decrease of SPINA-GD after glucose load only in PCOS women, the change was only minor. There are some possible explanations of this small decline in SPINA-GD during OGTT. First of all, it could be the result of low sensitivity of serum hormones concentration measurements. Most importantly, it is well known that the production or inactivation of T3 depend on different expression of DIOs in suitable organs, e.g., thyroid gland, as well as extrathyroid tissues: white adipose tissue, brown adipose tissue, muscle, central nervous system, liver, kidneys and skin [22]. Therefore, the estimated level of serum fT3 is derived from several sources, like formation and deiodination in the thyroid gland and deiodination in many peripheral organs. Moreover, it has been shown that plasma is the source of the largest extrathyroidal amount of T4, although approximately two-thirds of all T3 is found in the intracellular space in different tissues. Accordingly, T3 accumulated in extrathyroidal tissues could originate from two different sources: plasma T3 and local deiodination of T4 [22]. In fact, the estimation of serum concentration of T3 does not exactly show the amount of T3 in all intracellular compartments. Therefore, the decrease in serum concentration of T3 might not exactly reflect tissue T3 concentration after glucose load. On the other hand, it has been shown that plasma T3 equilibrates rapidly with most tissues [22].

Additionally, we observed a positive relationship between delta fT3 and plasma TG concentration, delta GD and plasma TG concentration after glucose load, and negative relationship between SPINA-GD2 and plasma TC concentration after glucose load only in PCOS women.

As mentioned in the Introduction section, TH influence many metabolic effects [6]. The impact of TH on atherosclerotic CVD may be partially explained by thyroid hormone regulation of lipid metabolism. The current knowledge assumes that mainly T3 exerts biological effect on lipid metabolism. In the face of the fact that in the present study we observed lower conversion of fT4 to fT3 and relationships of SPINA-GDs with TC and TG after OGTT in euthyroid PCOS women, we hypothesized that it could lead to atherosclerosis in young PCOS women. There is a concept that low-normal thyroid function, i.e., higher serum concentration of TSH and/or lower serum fT3, fT4 concentrations even within the reference range could exert a negative impact on CVD [23]. It has been published that mice with disrupted DIOs gene promote carbohydrates as a fuel source and have limited ability to mobilize and to burn fat. They are therefore predisposed to increased fat storage in adipose tissue, hepatic steatosis, and increased gluconeogenesis [24]. Akarsu et al. estimated DIOs in different metabolic states and observed that subcutaneous adipose tissue DIO2 gene expression is reduced in obese individuals with metabolic syndrome and negatively correlated with serum TG concentration [25].

The possible explanation of reduced deiodinase activity after glucose load in PCOS women could be connected with genetic factors, e.g., specific polymorphisms in the deiodinase genes (DIO1 and DIO2) [26–28]. It has been shown that a common variant rs225014 (c.274A > G, p.Thr92Al) of the gene encoding DIO2 is associated with reduced enzyme activity, as well as insulin resistance estimated with HOMA-IR and type 2 diabetes [26, 27]. Moreover, researchers postulated that lower DIO2 activity causes a decrease in DIO2-generated T3 concentration in skeletal muscle, which is connected with relative intracellular hypothyroidism. The next step is the reduction in the expression of different genes, for example glucose transporter 4 (GLUT4), leading to insulin resistance [27]. Moreover, the polymorphic variant c785C > T of the DIO1 gene results in decreased serum T3 concentrations, as well as is associated with elevated serum insulin growth factor (IGF-1) concentrations [29], whereas carriers of the D1a-T allele had higher serum fT4 and rT3 and lower T3 concentration [30]. Therefore, genetic background of decreased SPINA-GD2 should be taken into consideration in PCOS women. However, in our study we did not perform genetic analysis.

The results obtained in our study could be also explained by type 1 and type 2 thyroid allostasis [31]. Thyroid allostasis is an adaptive reaction of thyrotrophic feedback control system under a variety of developmental and straining conditions [31, 32]. It explains various adaptive processes starting in fetal life, starvation, strenuous exercise, depression, and drug effects (type 1 allostasis), as well as obesity, pregnancy, endurance exercise, acute psychosis, drug effects, post-traumatic reactions, and stress disease (type 2 allostasis) [31]. In type 1 thyroid allostatic load, serum concentration of T3 is downregulated, whereas serum level of T3 is upregulated in type 2 thyroid allostatic load. In situations of inflammation, common in metabolic syndrome, a variety of mediators (including tumor necrosis factor-α, interleukin 6, and interleukin 10) could have an impact on NF-κB pathway. It has been shown that NF-κB/IL6 signaling pathway inhibits T3-induced expression of DIO1, which results in reduced concentrations of free and total T3 [31]. Obesity, on the other hand, is a result of type 2 allostatic load. Therefore, it is possible that low grade chronic inflammation present in PCOS results in type 1 thyroid allostasis after glucose load in PCOS women. Moreover, an overlap of type 2 allostasis resulting from obesity and type 1 allostasis due to inflammation [31] could well explain the somewhat paradoxical pattern of slightly higher SPINA-GD1 in the PCOS group and more pronounced reduction in deiodinase activity after OGTT in PCOS women. Additionally, long-term effects of type 2 allostatic load include endothelial cell damage, hypertension, dyslipidemia, obesity, and type 2 diabetes mellitus [31], are often present in PCOS women.

In the present study we also observed decreased plasma concentration of TC, TG, HDL-C, and LDL-C after OGTT in women with PCOS, as well as in the control group. These findings are consistent with other researchers who found that plasma concentrations of TC, LDL-C, as well as remnant-like particles—cholesterol, apolipoprotein B, and small dense low-density lipoprotein (sdLDL-C), significantly decreased during OGTT [33]. The authors emphasized the fact that the change of sdLDL-C during OGTT had an inverse relationship with serum insulin level. Therefore, the researchers concluded that insulin can be one of the key modulators of serum sdLDL-C level, as well as LDL-C metabolism. It should be mentioned that in our study we found a positive relationship between HOMA-IR and serum TG concentration and negative association with the plasma level of HDL-C in PCOS. Therefore, insulin resistant state observed in PCOS women could predispose to atherogenic profile in this syndrome.

We examined young and non-obese women with PCOS characterized by lower insulin sensitivity and higher level of glucose and insulin concentration at the 60 min of OGTT in comparison to the control group. In the face of the fact that we found a relationship between insulin at the 60 min of OGTT and delta GD, we speculated that a postprandial decrease in the activity of peripheral DIOs could be connected with increased glucose and then insulin concentrations during OGTT in PCOS women. Although many studies focus on explaining the mechanism involved in the regulation of glucose homeostasis by TH, it still remains unclear. Nonetheless, the available studies showed impact of T3 on glucose metabolism. Ortega et al. showed that serum fT3 concentration is connected with fasting plasma insulin concentration and acute insulin response in euthyroid healthy adults with normal glucose tolerance test [34]. It has been shown that injection of exogenous T3 induces insulin-stimulated glucose transport by increasing GLUT4, and that T3 increased glycolysis in rat skeletal muscle [35]. Moreover, T3 administration decreased blood glucose level within 2 h and increased insulin sensitivity, insulin synthesis and storage in pancreatic beta cells, as well as increased plasma insulin level in obese mouse model of type 2 diabetes [36]. On the other hand, in the experimental model, it has been observed that in hypothyroidism glucose uptake in adipose tissue and in muscle is resistant to insulin, resulting in elevated serum insulin concentration [37]. So far, there is no observation about the decrease in serum fT3 concentration after glucose load in PCOS women. The impact of this finding on metabolism is not fully elucidated. Nevertheless, decreasing fT3 concentrations during OGTT could be connected with glucose and insulin disturbances. We can hypothesize that decreased intracellular conversion of the prohormone T4 to its active metabolite T3 could lead to decreased transcription rate of GLUT 4 in skeletal muscle and adipose tissue and cause impaired insulin-stimulated glucose disposal. However, the explanation of decreasing DIOs during OGTT in the PCOS group is unclear.

In our study, we observed decreased serum TSH concentration during OGTT in both studied groups. We observed that at baseline and at the 120 min of OGTT, TSH were within normal range. Our results are consistent with other researchers [38]. As the authors discussed, it could be connected with the stimulation of somatostatin release from the hypothalamus by hyperglycemia, thus causing inhibition of growth hormone, as well as TSH secretion.

In our study, we observed a relationship between SPINA-GD1 and BMI only in PCOS women. Moreover, we also noticed an association between fT3/fT4 ratio and BMI in this group. Our results are confirmed by other data collected in general euthyroid population [39, 40]. In a study conducted by Kitahara et al., a positive relationship between BMI, waist circumference, and fT3 among euthyroid subjects has been found [40]. In another cross-sectional study, a relationship between BMI and fT3 in a cohort of euthyroid overweight and obese women has been confirmed [39]. In the research, performed before and after weight loss resulting from laparoscopic gastric banding, it has been shown that serum concentration of fT3 was higher in obese than in non-obese subjects before intervention and decreased after weight loss in obese subjects, while fT4 increased and TSH remained stable [41]. There is a concept that elevated serum fT3 in obesity is responsible for increased resting energy expenditure as a prevention of further fat accumulation [42].

The main limitation of the present study is a relatively small sample size, especially regarding the control group.

## Conclusions

On the basis of the obtained results, we concluded that decreased deiodinase activity after glucose load might influence plasma lipids and thus could promote atherosclerosis in PCOS women.
